# Optimal Protein Intake in Pre-Dialysis Chronic Kidney Disease Patients with Sarcopenia: An Overview

**DOI:** 10.3390/nu13041205

**Published:** 2021-04-06

**Authors:** Yoshitaka Isaka

**Affiliations:** Department of Nephrology, Osaka University Graduate School of Medicine, Suita 565-0871, Japan; isaka@kid.med.osaka-u.ac.jp; Tel.: +81-6-6879-3857; Fax: +81-6-6879-3230

**Keywords:** malnutrition, protein energy wasting (PEW), sarcopenia

## Abstract

Multi-factors, such as anorexia, activation of renin-angiotensin system, inflammation, and metabolic acidosis, contribute to malnutrition in chronic kidney disease (CKD) patients. Most of these factors, contributing to the progression of malnutrition, worsen as CKD progresses. Protein restriction, used as a treatment for CKD, can reduce the risk of CKD progression, but may worsen the sarcopenia, a syndrome characterized by a progressive and systemic loss of muscle mass and strength. The concomitant rate of sarcopenia is higher in CKD patients than in the general population. Sarcopenia is also associated with mortality risk in CKD patients. Thus, it is important to determine whether protein restriction should be continued or loosened in CKD patients with sarcopenia. We may prioritize protein restriction in CKD patients with a high risk of end-stage kidney disease (ESKD), classified to stage G4 to G5, but may loosen protein restriction in ESKD-low risk CKD stage G3 patients with proteinuria <0.5 g/day, and rate of eGFR decline <3.0 mL/min/1.73 m^2^/year. However, the effect of increasing protein intake alone without exercise therapy may be limited in CKD patients with sarcopenia. The combination of exercise therapy and increased protein intake is effective in improving muscle mass and strength in CKD patients with sarcopenia. In the case of loosening protein restriction, it is safe to avoid protein intake of more than 1.5 g/kgBW/day. In CKD patients with high risk in ESKD, 0.8 g/kgBW/day may be a critical point of protein intake.

## 1. Introduction

**Figure 1:**
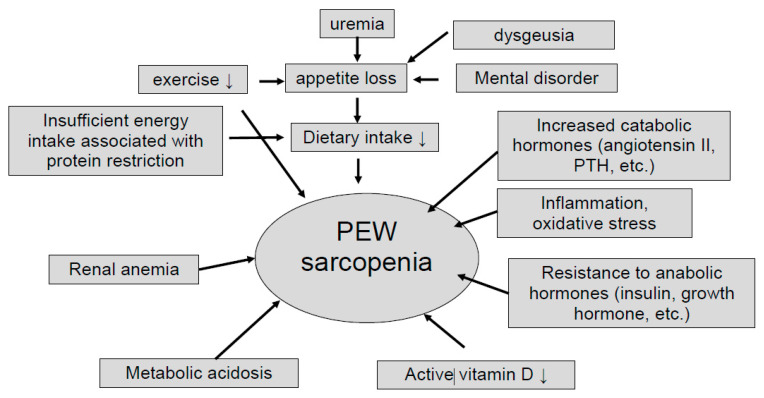
Potential causes of protein-energy wasting and sarcopenia. PEW; protein energy wasting, PTH; parathyroid hormone.

Nutritional adverse derangement is often observed in patients with chronic kidney disease (CKD), and common in advanced CKD and dialysis patients. The International Society of Renal Nutrition and Metabolism (ISRNM) defines protein energy wasting (PEW) as a “the state of decreased body protein and fat masses” [1]. The pathogenesis of PEW in CKD is multifaceted. Decreased protein and energy intake due to dietary restriction or anorexia, increased protein catabolism due to activation of renin-angiotensin system or hyperparathyroidism, decreased anabolism due to insulin resistance, chronic inflammation, metabolic acidosis, and hormonal imbalances have been reported to be associated with PEW [2] as well as sarcopenia [3] (Figure 1). These two concepts share the same criteria and have similar causes and outcomes, but they are defined differently [4]. PEW focuses on protein and energy loss associated with inflammation, whereas sarcopenia focuses on muscle mass and strength loss associated with aging. CKD patients often belong to both conditions to varying degrees.

Dietary protein intake gradually decreases during the progression of kidney injury, even in the CKD patients with minimal dietary intervention [5]. This trend was similarly observed for urinary creatinine excretion, a marker of muscle mass [5]. Food intake progressively and spontaneously decreases with decline of renal function [6]. Infusion of angiotensin II in rats was shown to induce skeletal muscle wasting via proteolysis [7,8], and in turn, angiotensin converting enzyme inhibitor [9] or angiotensin receptor blocker [10] preserved muscle strength. Parathyroid hormone (PTH) was reported to drive adipose tissue browning and malnutrition via PTH receptor in fat tissue [10]. Systemic inflammation, including elevated cytokines, such as tumor necrosis factor (TNF)-α, interleukin (IL)-6, IL-8, and so on, is often observed in CKD patients, and this tendency becomes more pronounced as the CKD stage progresses [11]. The inflammation in uremic milieu induces cardiovascular diseases and malnutrition [12,13]. Metabolic acidosis increases protein catabolism via up-regulation of ubiquitin-proteasome system in CKD patients [14,15]. Anemic patients were more frequently malnourished or at risk of malnutrition, and albumin levels are strongly associated with anemia in the elderly [16]. Vitamin D is associated with muscle weakness in older people [17], and pre-dialysis CKD patients [18].

As mentioned above, many factors contribute to malnutrition in CKD. Most of these factors, such as hyperparathyroidism, activation of renin-angiotensin system, metabolic acidosis, and insulin resistance, worsen as CKD progresses. Thus, nutritional condition in CKD patients deteriorates as CKD progresses. In fact, protein energy wasting (PEW) assessed by clinical global assessment was observed in 2% of CKD stage G1-2, 16% of CKD G3-4, 31% of CKD G5 without dialysis, and 44% of CKD G5D [19]. On the other hand, protein restriction is used as a treatment for CKD, but it may lead to sarcopenia, assessed by loss of muscle strength or mass [20]. Energy-adjusted protein intake was associated with three-year changes in lean mass body. Participants in the highest quintile of protein intake lost approximately 40% less lean mass than did those in the lowest quintile of protein intake [20].

As mentioned above, the more advanced CKD becomes, the more severe the malnutrition becomes. Inflammation and other conditions that cause PEW become more serious as CKD progresses. On the other hand, protein intake and exercise therapy are factors that can intervene in CKD patients with sarcopenia. In CKD patients with sarcopenia, increasing protein intake may prevent worsening of sarcopenia and improve life expectancy. On the other hand, increased protein intake may accelerate the progression of CKD. Considering these two opposing effects, it is important to consider the appropriate protein intake for CKD patients with sarcopenia. As the other section in this issue describes the pathogenesis of malnutrition in dialysis patients, this paper will focus on the optimal protein intake and exercise on pre-dialysis CKD patients with sarcopenia.

## 2. Definition and Epidemiology of Sarcopenia in CKD

Sarcopenia is a syndrome characterized by a progressive and systemic loss of muscle mass and strength, which is associated with physical dysfunction, poor quality of life, and risk of death. It is diagnosed when the loss of muscle mass is accompanied by a loss of muscle strength or physical performance [21]. In addition to common risk factors such as aging and physical inactivity, inadequate protein intake as well as energy deficiency is thought to play a major role in the development and progression of sarcopenia in CKD patients. Furthermore, various factors, such as inflammation [22], metabolic acidosis [23], natural vitamin D deficiency [24], or diuretics treatment [25,26] may contribute to the development and progression of sarcopenia in CKD patients [3]. There are multiple diagnostic criteria for sarcopenia, which are not standardized internationally. The European Working Group on Sarcopenia in Older People (EWGSOP) definition is often used in Western countries [21], while the Asian Working Group on Sarcopenia in Older People (AWGS) [27] is recommended in Asian countries. Recently, the consensus of EWGSOP [27] and AWGS [28] was updated. Both consensus guidelines emphasize muscle strength by grip strength, allowing for diagnosis by family physicians and community health care settings that lack equipment to measure skeletal muscle mass, because low muscle strength can predict a worse outcome than low muscle mass [29]. For the assessment of skeletal muscle mass, many of the cutoffs are based on Dual Energy X-ray Absorptiometry (DEXA), while others are based on the bioelectrical impedance analysis (BIA) method. There have been reports comparing the incidence of sarcopenia in CKD patients according to each cutoff value by using BIA, central upper arm circumference and subcutaneous fat, and subjective global assessment, and it has been reported that the incidence varies depending on the assessment method [25,30,31,32]. Reports on the epidemiological frequency of complications are currently scarce for any of the diagnostic criteria, and there is a wide range among reports for the same diagnostic criteria. Therefore, we need to choose the diagnostic criteria by considering the body size or race to diagnose sarcopenia.

According to the National Health and Nutrition Examination Survey (NHANES III, 1994–1998), CKD patients have a high incidence of muscle mass loss [33]. In addition, a cohort study showed that walking speed and muscle strength decreases as the CKD stage progressed from creatinine clearance (Ccr) of 90 mL/min or more to 60–89 mL/min and less than 60 mL/min [34]. In a study of CKD patients with minimal dietary guidance, patients with lower Ccr had lower protein intake and lower urinary creatinine excretion, suggesting that appetite decreases and muscle mass decreases as CKD progresses [5]. According to the Korea National Health and Nutrition Examination Survey, the frequency of sarcopenia increases with CKD stage, with 2.6%, 5.6%, and 18.1% of men and 5.3%, 7.1%, and 12.6% of women in CKD stages 1, 2, and 3–5, respectively [30]. Thus, the frequency of sarcopenia is higher in CKD patients than in the general population, and it increases with the progression of CKD stage.

The prognosis of CKD complicated by sarcopenia is worse than that of uncomplicated CKD in terms of mortality and length of hospital stay. In a report examining creatinine excretion and mortality risk, the risk of mortality increases with decreasing creatinine excretion and decreasing muscle mass [35]. In patients with stage 3–5 CKD diagnosed with sarcopenia assessed by BIA, by central upper arm circumference and subcutaneous fat, and by subjective comprehensive assessment, sarcopenia is associated with poor prognosis regardless of the diagnostic method [31]. Furthermore, in hemodialysis patients, the diagnosis of sarcopenia by grip weakness or definition of EWGSOP is associated with mortality risk [32]. In a report on hemodialysis patients from Japan, the modified creatinine (Cr) index using pre-dialysis serum Cr, which is correlated with muscle mass, was assessed by the BIA method, and the lower this value is, the higher the risk of fracture [36].

## 3. CKD with Sarcopenia and Protein Restriction

End-stage kidney disease (ESKD) and death/cardiovascular death (mortality) are both important as the outcomes of CKD patients, and protein restriction is mainly used to improve the outcome of the former. In elderly CKD patients, the mortality risk is higher than the risk of ESKD [37]. Many of CKD patients with sarcopenia have been treated with protein restriction as a standard therapy. Kidney Disease Quality Initiative—National Kidney Foundation (KDOQI-NKF) guidelines for nutrition in CKD recommends a protein intake of 0.6 to 0.8 g/kg/day for patients with CKD in stages 3 to 5 with an energy intake of 30 kcal/kg/day [38]. The PROT-AGE Study Group recommends a protein intake of 0.8 g/kg/day and >0.8 g/kg/day for the elderly CKD patients with GFR < 30 mL/min and 30 to 60 mL/min, respectively [39]. However, in the case of muscle wasting such as sarcopenia, sufficient energy (30 kcal/kg/day) and protein (0.8–1.0 g/kg/day) are recommended for nutritional needs [4]. Some CKD patients with sarcopenia are at high risk for CKD progression to end-stage renal failure, while others have worsening sarcopenia and are at high risk of shortened life expectancy. In CKD patients with sarcopenia, different decisions (protein intake or protein restriction) need to be made against the dual outcomes of the progression of renal damage and the progression of sarcopenia. If the risk of end-stage renal failure is high, protein restriction is necessary, and if the risk of worsening sarcopenia is high, protein restriction should be loosened. However, such criteria are not clear. Thus, it is important to decide whether protein restriction should be continued or loosened in CKD patients with sarcopenia. CKD patients classified to stage G4 to G5 are belong to the extremely high risk group of renal replacement therapy, and prone to complications such as accumulation of uremic toxins, electrolyte abnormalities, and metabolic disorders [40,41]. Protein restriction can reduce the risk of ESKD in CKD, especially in patients with a GFR < 30 mL/min/1.73 m^2^, but does not increase the risk of death [42], suggesting that protein restriction should be considered a priority for patients with CKD stage G4 to G5.

The relative risk of ESKD for CKD patients with stage G3 varies greatly depending on the urinary protein level and rate of eGFR decline. Therefore, the risk of ESKD should be assessed in each individual case to determine whether protein restriction should be continued or loosened in CKD patients with stage G3. It has been reported that the risk of ESKD is low in CKD patients with A1 and A2 severity categories, and the mortality risk is higher than the risk of ESKD in cases with urinary protein levels <0.5 g/day [43]. On the contrary, it has been reported that proteinuria >1.0 g/gCr [44] or albuminuria > 1.0 g/gCr [45] is associated with a higher risk of ESKD.

With regard to the rate of eGFR decline, it has been reported that CKD patients with an eGFR decline >3.0 mL/min/1.73 m^2^/year have a higher risk of ESKD than those with a lower eGFR decline [46,47]. In a meta-analysis showing that protein restriction suppresses the rate of GFR decline [48], 12 of the 15 studies showed that the rate of GFR decline was greater than 3.0 mL/min/1.73 m^2^/year, suggesting that protein restriction may be effective in patients with a faster rate of eGFR decline. Thus, it is reasonable to consider prioritizing protein restriction in CKD patients with stage G4 to G5, but loosening protein restriction in CKD stage G3 patients with proteinuria <0.5 g/day, and rate of eGFR decline <3.0 mL/min/1.73 m^2^/year.

## 4. Effect of Increased Protein Intake for CKD Patients with Sarcopenia

The supplementation of vitamin D and leucine-enriched diet in elderly patients with sarcopenia for 13 weeks improved the chair-rise test and limb muscle mass compared with controls, but there was no difference in grip strength or short physical performance battery (SPPB) [49]. On the other hand, in a study of sarcopenic older adults using nutritional supplements with different amounts of protein, lower extremity muscle strength, muscle quality, grip strength, and walking speed increased in high protein intake groups after 24 weeks [50]. In addition, in a randomized controlled trial (RCT) of protein loading in elderly people with frailty, protein intake improved short physical performance battery (SPPB) but not lean mass [51]. As described above, there are a number of RCTs showing the efficacy of dietary therapy alone, but none of them reported that dietary therapy was effective for skeletal muscle mass, physical function, or muscle strength. In a meta-analysis of five RCTs of elderly people diagnosed with sarcopenia, there was no clear effect on skeletal muscle mass, lean mass, grip strength, knee extensor strength, walking speed, or Timed Up and Go test at three months [52]. Thus, the effect of increasing protein intake alone without exercise therapy may be limited in CKD patients with sarcopenia.

## 5. Increased Protein Intake and Exercise for CKD Patients with Sarcopenia

In a systematic review and meta-analysis of three RCTs of elderly people with sarcopenia, exercise therapy improves limb skeletal muscle mass, normal walking speed, maximal walking speed, and knee extension muscle strength compared with dietary intervention or health education [52], suggesting that exercise therapy is effective in improving sarcopenia. In addition, there are several reports that exercise therapy, including resistance exercise, prolonged six-minute walking distance [53], increases thigh cross-sectional area, volume, and knee extension muscle strength [54], and increases muscle fiber area and upper and lower limb muscle strength [55,56]. Furthermore, in a 12-week RCT in CKD patients with stages G3b to G5, including frailty patients, the combination of resistance exercise and aerobic exercise increases muscle mass and strength compared with aerobic exercise alone [57], and it is important to note that there are no significant changes in renal function in both groups. These results suggest that exercise therapy is effective in improving muscle mass and strength in elderly patients with sarcopenia, and that the combination of exercise therapy and diet therapy is more effective than exercise therapy alone in improving sarcopenia in elderly patients. On the other hand, a large increase in protein intake may worsen the renal function in CKD patients. Therefore, it is considered safer to increase protein intake gradually in CKD. Although the amount of energy consumed during exercise therapy varies widely among individuals, total energy requirements are also expected to increase, so energy intake should be adjusted accordingly.

## 6. Excessive Protein Intake in CKD Patients

The GFR increases physiologically and transiently with protein intake. In the elderly, especially those over 70 years of age, potential glomerular hyperfiltration occurs due to age-related nephron loss [58], and hypertension, diabetes mellitus, and obesity further reduce renal reserve [59]. It has been reported that short-term protein loading (average of 2.0 g/kgBW/day) increases GFR in healthy young adults, but decreases GFR in older adults (average of 1.8 g/kgBW/day for 10 days) [60]. In a report from the United States on healthy subjects with an eGFR > 60 mL/min/1.73 m^2^ without cardiovascular disease or diabetes for a median of 23 years [61], a report from the Netherlands on healthy subjects with a mean eGFR of 80 mL/min/1.73 m^2^ for a mean of 6.4 years [62], and a report from the United States on healthy women with an eGFR > 80 mL/min/1.73 m^2^ for 11 years [63], protein intake was not associated with decreased renal function. Thus, in terms of risk of renal function decline, CKD stage G1 to G2 patients with sarcopenia may benefit from adequate dietary protein intake. On the other hand, an association between high protein intake and cardiovascular mortality has been reported in elderly people at risk for cardiovascular disease [64]. In a Spanish report of elderly subjects at high risk for cardiovascular disease, the risk of cardiovascular and all-cause mortality was higher in the group with a protein intake >1.5 g/kgBW/day compared with the group with a protein intake of 1.0–1.5 g/kgBW/day at a median observation period of 4.8 years [64], suggesting that protein intake may be an independent risk factor for cardiovascular disease risk. Therefore, it is safe to avoid protein intake of more than 1.5 g/kgBW/day at least in elderly people at risk for cardiovascular disease.

In the MDRD (Modification of Diet in Renal Disease) Study A, there was no difference in glomerular filtration rate (GFR) reduction between usual-protein diet group (1.3 g/kg body weight (BW)/day) and low-protein diet group (0.58 g/kgBW/day) among CKD patients (eGFR 25–55 mL/min/1.73 m^2^) over the entire three-year analysis [65]. In a subsequent analysis, the rate of GFR decline in the 0.58 g/kgBW/day group was faster than that in the 1.3 g/kgBW/day group up to four months after the start of the study, and the rate of GFR decline was slower in the 0.58 g/kgBW/day group after four months, suggesting the possibility of a long-term renoprotective effect of a low-protein diet [66]. In an RCT of 89 patients with CKD stage G3 and hypertension [67], the GFR decline at 12 months was faster in the unrestricted group (actual intake: 1.54 ± 0.39 g/kgBW/day) compared with the patients with a protein intake instruction of 0.6 g/kgBW/day (actual intake: 0.67 ± 0.21), suggesting that the actual intake of 1.5 g/kgBW/day of protein worsens the rate of renal function decline compared with 0.6 g/kgBW/day. In this report, serum albumin and prealbumin levels did not change in the 0.6 g/kgBW/day group, but energy intake, body weight, and BMI decreased, compared with the unrestricted group. A French report of CKD (stage G3: 50%) with a median follow-up of 5.6 years showed that an increase in protein intake of 0.1 g/kgBW/day increased the risk of ESKD by 1.05 (95% CI, 1.01–1.10) [68]. However, the hazard ratio was even higher in the group with GFR < 30 mL/min/1.73 m^2^, but the significance disappeared in the group with GFR ≥ 30 mL/min/1.73 m^2^, suggesting that the effect of protein restriction in stage G3 is not high [68]. Furthermore, the risk of ESKD increases linearly with increasing protein intake, but there is no threshold for protein intake [68]. Thus, the upper limit of protein intake for CKD patients who are not at high risk for ESKD is 1.3 g/kgBW/day under the presence of sarcopenia.

On the other hand, protein restriction should be prioritized in CKD patients at high risk for ESKD. CKD patients prioritizing protein restriction are considered to be stage G4-G5 patients and stage G3 patients with proteinuria >0.5 g/day. However, excessive protein restriction may exacerbate sarcopenia. A systematic review and meta-analysis of RCTs in stage G3 to G5 CKD reported that protein restriction of <0.8 g/kgBW/day was associated with a reduced risk of progression to ESKD compared with >0.8 g/kgBW/day, with no change in the risk of total mortality [69]. In an RCT of CKD in stages G4 to G5 with strict protein restriction (0.55 g/kgBW/day) versus usual protein restriction (0.8 g/kgBW/day), there was no difference in survival, non-induction of dialysis, or their combined outcomes [70], suggesting that protein restriction of 0.8 g/kgBW/day does not further worsen renal dysfunction compared to 0.55 g/kgBW/day. Thus, 0.8 g/kgBW/day is considered to be a critical point of protein intake in CKD patients with high risk in ESKD.

## 7. Conclusions

Reflecting the aging of CKD patients in recent years, malnutrition and sarcopenia have been the focus of much attention. Although many factors are thought to be involved in the development of these pathogenesis, inadequate protein intake may contribute to the progression of sarcopenia. Protein restriction has been used to treat CKD patients for many years, and protein intake for CKD patients with sarcopenia may improve sarcopenia and improve life expectancy. On the other hand, excessive protein intake may accelerate the progression of CKD. For CKD patients with sarcopenia, urinary protein excretion and the rate of eGFR decline should be evaluated to determine whether protein restriction should be continued or loosened.
